# Diagnosis of Awake Bruxism in Adolescents and Young Adults: A Systematic Review

**DOI:** 10.3390/dj14090606

**Published:** 2026-09-18

**Authors:** Javier García-Moya, Juan Antonio Ruiz-Roca, María Pilar Pecci-Lloret, Alberta Lucchese, Dorina Lauritano, Nuria Pérez-Guzmán

**Affiliations:** 1 Dermatology, Estomatology, Radiology and Physical Medicine, University of Murcia, 30008 Murcia, Spainmariapilar.pecci@um.es (M.P.P.-L.); nuria.perez5@um.es (N.P.-G.); 2 Multidisciplinary Department of Medical-Surgical and Dental Specialties, University of Campania “Luigi Vanvitelli”, 81100 Naples, Italy; alberta.lucchese@unicampania.it; 3 Department of Translational Medicine, University of Ferrara, 44121 Ferrara, Italy; dorina.lauritano@unife.it

**Keywords:** awake bruxism, adolescents, diagnosis, ecological momentary assessment, electromyography, systematic review

## Abstract

**Background/Objectives**: Awake bruxism is a masticatory muscle activity occurring during wakefulness and expressed as tooth contact, clenching, grinding or mandibular bracing. Its diagnosis in adolescents and young adults remains controversial due to the absence of a universally accepted gold standard. This systematic review aimed to assess the diagnostic methods used for awake bruxism in this population. **Methods**: This systematic review followed PRISMA 2020 guidelines and was registered in PROSPERO. Searches were performed in PubMed/MEDLINE, SciELO, Scopus, Cochrane Library and Web of Science on 17 March 2026. Studies assessing diagnostic or screening methods for awake bruxism in adolescents and young adults under 35 years were included. Risk of bias was assessed using JBI, RoB 2 and QUADAS-2 tools. **Results**: From 418 records identified, 13 studies met the inclusion criteria. After excluding two studies with high risk of bias, 11 were included in the qualitative synthesis. The main diagnostic approaches were self-report questionnaires, ecological momentary assessment, clinical examination and portable electromyography. Self-report was useful for screening but showed limited reliability. Ecological momentary assessment improved real-time behavioural recording, while clinical signs provided complementary information. Portable electromyography provided a more objective masticatory muscle activity, although standardized diagnostic thresholds are still lacking. **Conclusions**: No gold standard currently exists for diagnosing awake bruxism in adolescents and young adults. A multimodal approach combining self-report, ecological momentary assessment, clinical examination and electromyography appears to be the most robust diagnostic strategy.

## 1. Introduction

According to the International Consensus on the Evaluation of Bruxism, bruxism is defined as a repetitive masticatory muscle activity characterised by biting and/or grinding of the teeth at different times of the day, such as during sleep (sleep bruxism) (SB) and while awake (awake bruxism) (AB) and/or jaw immobilisation or thrusting. In healthy individuals, it is not considered a disorder, but it can be a risk factor for negative consequences for oral health [1]. This is a repetitive or sustained contact of the teeth and/or sustained stiffness of the masticatory muscles with forced jaw movement, to the sides or forward, during the day [2].

Persistent awake bruxism (AB) behaviours can exert excessive functional forces on the dentition and associated oral structures [3]. When sustained over time, these behaviours may contribute to several adverse consequences for the stomatognathic system, including dental attrition, temporomandibular joint dysfunction, headaches, and fatigue, pain, and a reduced pain threshold in the masticatory and cervical muscles [4]. In addition, these clinical manifestations may negatively affect oral health-related quality of life.

The global prevalence of bruxism has been estimated at 22.2%, while the awake bruxism is about 23.29% (95% CI: 18.78–28.52%), suggesting that approximately one in four individuals may experience awake bruxism [5].

According to the meta-analysis by Zieliński et al. in 2024 [5], the prevalence of SB and AB in Europe is 21% and 18%, respectively, much lower than in North America, where it was 31% and 30%, respectively. These data also tell us that both types of bruxism occur almost equally in the population. The same study found that there are no statistically significant differences in AB with regard to gender, and with regard to age, the prevalence in European adults is notably higher at 22.55% compared to European minors at 16.17%. North American data show higher prevalence rates, particularly among adults at 36.39% compared to 28% in minors [5].

While there are numerous objective diagnostic methods and instruments available for sleep bruxism (SB), such as polysomnography and electromyography (EMG) [6], the assessment of awake bruxism (AB) can involve a range of approaches, from clinical examination to tools such as BruxApp, BruxScreen, and BruxChecker, as well as questionnaires including the Research Diagnostic Criteria for Temporomandibular Disorders (RDC/TMD), Diagnostic Criteria for Temporomandibular Disorders (DC/TMD), the Oral Behavior Checklist (OBC), and the Pittsburgh Sleep Quality Index (PSQI) [5]. Instrumentally driven assessment of awake bruxism, based on the principles of the Ecological Momentary Assessment (EMA), has shown that a certain amount of bruxism-related masticatory muscle activity can be expected in all individuals, and that the clinical relevance is related to the frequency and intensity of the events. Furthermore, the advent of portable surface electromyographic (EMG) devices that can record 24 h jaw muscle activity has provided new possibilities to monitor both AB and sleep bruxism in the natural environment [7]. The present systematic review aimed to evaluate the available diagnostic tools for assessing awake bruxism in adolescents and young adults.

## 2. Materials and Methods

This systematic review study followed the recommendations of PRISMA 2020 (Table S1) and PROSPERO with register number CRD420251181371.

### 2.1. Search Strategy

An electronic search was conducted on 17 March 2026, across different databases: PubMed (Medline), SciELO, Scopus, Cochrane and Web of Science. The study selection, data extraction and risk of bias analysis was carried out by MPPL and NPG; in case of discrepancies, a third reviewer was asked (JARR).

### 2.2. Search Terms

The search strategy used across all databases were as follows:

(“awake bruxism” OR “waking bruxism” OR “diurnal bruxism”)

AND (child* OR adolescent* OR pediatric OR paediatric OR “young adult*” OR youth OR teen*) AND (diagnos* OR assessment OR screening OR identification OR criteria)

### 2.3. Article Selection and Eligibility Criteria

The research question was formulated according to the PECOS framework, as follows: Population: adolescents and young adults (<35 years old) patients; Exposure: use of different methods to assess awake bruxism; Comparison: between assessments methods (when applicable); Outcome: correlation of the method and awareness of the habit with the method.

Studies were included if they involved adolescents or young adults (<35 years) and evaluated a method for the assessment, screening, or diagnosis of awake bruxism. Original observational, diagnostic accuracy, and interventional studies were considered.

Studies were excluded if they focused exclusively on sleep bruxism, investigated bruxism only as a secondary variable (like prevalence or associated factors), or did not describe a clear assessment method. Review articles and theses were also excluded.

### 2.4. Assessment of Risk of Bias

Risk of bias was assessed using design-specific tools selected according to the methodological design of each included study. Cross-sectional studies were evaluated using by the Joanna Briggs Institute (JBI) Critical Appraisal Checklist for cross-sectional studies [8], randomised trials using the Revised Cochrane Risk of Bias Tool for Randomized Trials (RoB 2) [9], and diagnostic accuracy studies using the Quality Assessment of Diagnostic Accuracy Studies-2 (QUADAS-2) tool [10]. The risk-of-bias assessment was independently performed by two reviewers (MPPL and NPG). Any disagreements were resolved through discussion and, when consensus could not be reached, by consultation with a third reviewer (JARR).

Studies judged to be at high risk of bias were excluded from the qualitative synthesis in order to minimise the influence of substantial methodological limitations on the interpretation of the findings and to ensure that the conclusions were based on studies with an acceptable level of methodological quality.

A formal GRADE assessment of the certainty of evidence was not performed because of the substantial methodological heterogeneity among the included studies, particularly regarding study design, diagnostic approaches, outcome measures, recording protocols, and diagnostic thresholds, which precluded the identification of sufficiently homogeneous bodies of evidence for specific diagnostic outcomes.

## 3. Results

### 3.1. Article Selection and Flow Diagram

**Figure 1:**
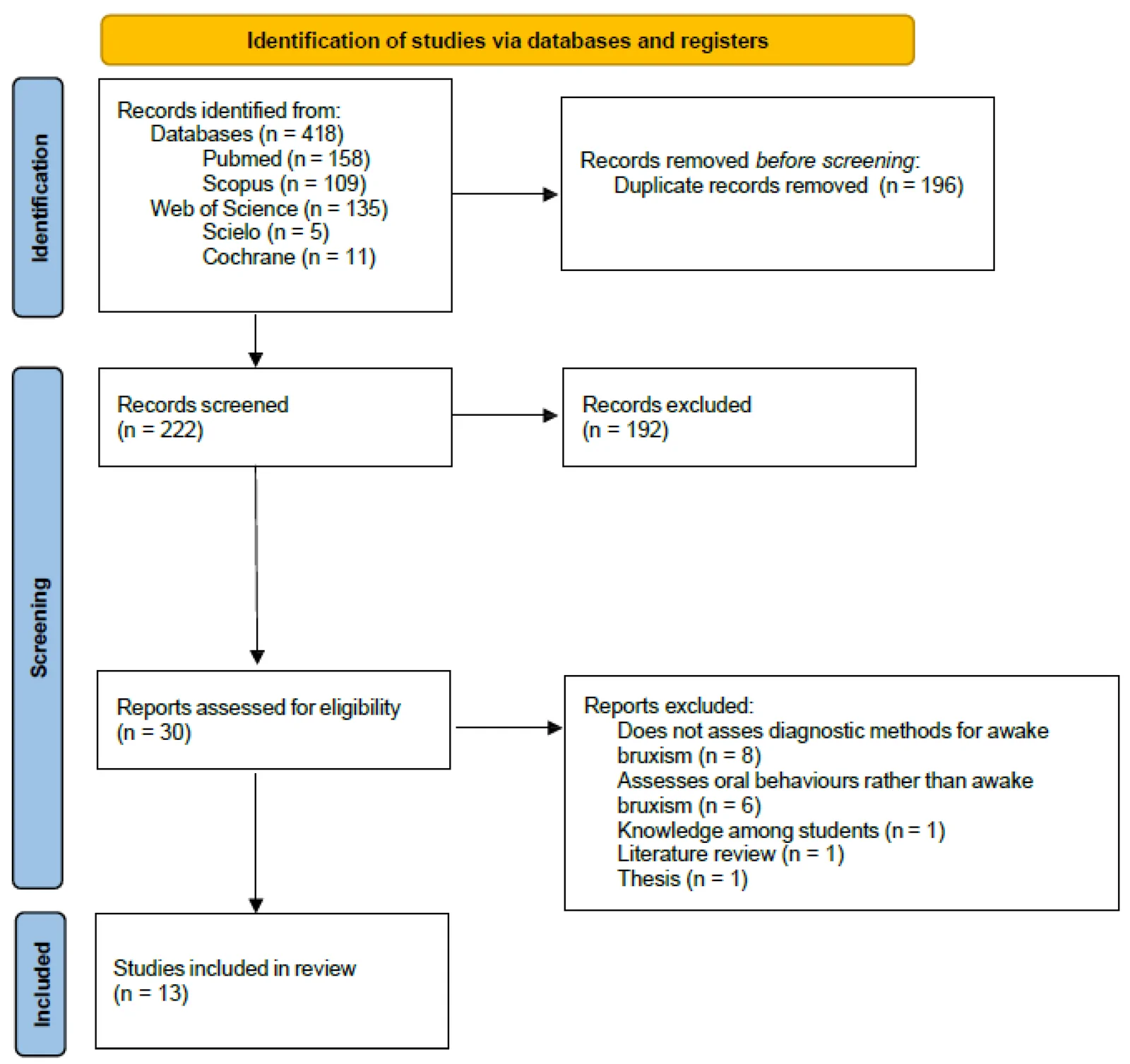
Flow diagram PRISMA 2020.

After obtaining 418 articles from five databases (PubMed = 158, Scopus = 109, Web of Science = 135, SciELO = 5, Cochrane = 11), the duplicate records were removed before screening (196), leaving a total of 222 records screened. After title and abstract screening, 192 records were excluded and 30 reports were assessed for eligibility. From the 30 reports, 17 were excluded, eight did not assess diagnostic methods for awake bruxism, six assessed oral behaviours rather than awake bruxism, one was about knowledge among students, one was a literature review and one was a thesis. Finally, 13 articles met the inclusion criteria as seen in Figure 1, which summarises the study selection process according to the PRISMA 2020 flow diagram.

### 3.2. Quality Assessment—Risk of Bias

The JBI checklist was used to determine the risk of bias of cross-sectional studies (Table 1). Of the thirteen articles, two showed a high risk of bias and twelve showed a moderate risk of bias.

The risk of bias of one randomised interventional study was assessed using the RoB 2 tool (Table 1), and this article showed a high risk of bias [11].

The methodological quality of diagnostic accuracy study was assessed using the QUADAS-2 tool (Table 1) and this article showed a moderate risk of bias [12].

A total of 13 studies met the eligibility criteria and were included in the systematic review. Following the risk-of-bias assessment, two studies were judged to have a high risk of bias and were excluded from the qualitative synthesis. Consequently, 11 studies were included in the final analysis.

**Table 1:** Risk of bias.

JBI Cross-Sectional									
Author	1	2	3	4	5	6	7	8	Overall
Colonna et al. 2020 [13]	Y	Y	P	P	N	N	P	Y	Moderate
Colonna et al. 2025 [14]	Y	Y	P	P	N	N	P	Y	Moderate
Dias et al. 2021 [15]	Y	Y	P	P	N	N	P	Y	Moderate
Flores et al. 2024 [16]	Y	Y	P	P	P	N	Y	Y	Moderate
Nakagawa et al. 2025 [17]	Y	Y	Y	P	P	N	Y	Y	Moderate
Prado et al. 2023 [18]	Y	Y	P	P	P	N	P	Y	Moderate
Saracutu et al. 2025 [19]	Y	Y	P	P	P	N	P	Y	Moderate
Schappo et al. 2023 [20]	Y	Y	P	P	N	N	P	Y	Moderate
Stanisic et al. 2024 [21]	Y	Y	P	P	P	N	P	Y	Moderate
Zani et al. 2019 [22]	P	P	P	N	N	N	P	P	High
Zani et al. 2021 [23]	Y	Y	P	P	N	N	P	Y	Moderate
**RoB 2**									
**Author**	**Dom 1**	**Dom 2**	**Dom 3**	**Dom 4**		**Dom 5**		**Overall**	
Dias et al. 2024 [11]	Some concerns	High risk	Low Risk	High risk		Some concerns		High risk	
**QUADAS-2 risk of bias**									
**Author**	**1**	**2**	**3**	**4**		**Overall**			
Angkulmahasuk et al. 2025 [12]	Some concerns	Some concerns	Some concerns	Low risk		Moderate			

*Note: Y: Yes N: No. P: Partial*.

### 3.3. Data Extraction Results

The characteristics of the 11 studies included in the qualitative synthesis are presented in separate tables according to the main diagnostic approach used, distinguishing between non-instrumental methods (EMA, questionnaires and clinical examination) in Table 2 and instrumental EMG-based assessment in Table 3.

Most of the studies were conducted in Europe, specifically four in Italy [13,14,19,22], one in Sweden [21] and another in Portugal [15]; followed by South America with three in Brazil [16,18,20]; and two in Asia, specifically in Japan [12,17].

Overall, the included studies showed marked methodological heterogeneity in the assessment of awake bruxism, particularly regarding the operational definition of bruxism, the recording period, and the diagnostic threshold used to classify participants.

The study by Stanisic et al. [21] included 10 young adults aged 23–30 years and 10 of their parents aged 42–67 years. In accordance with the predefined eligibility criteria of this systematic review, only data from the 10 young adult participants were extracted and considered in the present analysis; data from the parent group were not included.

Almost all studies refer to “possible” or “probable” bruxism and use Ecological Momentary Assessment (EMA), based on real-time self-reporting of awake bruxism behaviours, to assess it, while also recording five oral parameters or conditions to be assessed: relaxed masticatory muscles, dental contact, clenching, grinding, and jaw tension.

Two authors from South America, Prado et al. [18] and Schappo et al. [20], also include clinical signs and symptoms resulting from clinical examination, such as indentations on the tongue or the linea alba on the buccal mucosa. However, studies conducted in Japan [12,17] refer to definitive bruxism and, to this end, employ both a self-reported questionnaire, such as the OBC (Oral Behaviour Checklist), and electromyography (EMG) using a portable device. This device was the FLA-500-SD model (Furusawa Lab Appliance Co.), which consisted of a miniature recorder placed at the centre of the masseter muscle on the habitual chewing side, taking measurements for at least 8 h a day over three separate days, while performing normal daily activities and consuming at least two meals [12,17]. In addition, Angkulmahasuk et al. [12] added 15 min recordings while performing four specific tasks (silent reading, manipulating beads with chopsticks, watching relaxing videos and simple mathematical tasks).

Portable EMG provides the most direct measure of masticatory muscle activity during wakefulness; however, the absence of universally accepted cut-off points and the variability in recording protocols limited its comparability across studies.

Regarding the questionnaire for assessing bruxism, almost all [13,14,15,21,23] used the BruxApp^R^ mobile app, whose software sent 20 random alerts daily until 7 consecutive days of recording were completed, with the success threshold set at 60% of alerts responded to others [16,20]. However, Schappo et al. [20] and Medina Flores et al. [16] used WhatsApp to deliver EMA prompts, with 10 and 15 daily alerts, respectively, over 7 consecutive days. Participants were required to respond immediately or within a maximum of 10 min of receiving the message. The remaining, more traditional questionnaires consisted either of subjective dichotomous questions [17,18] or questionnaires such as the OBC [15], the Brux-5Q [21] and the Diagnostic Accuracy of Three Screening Questions 3Q/TMD in relation to the Diagnostic Criteria for Temporomandibular Disorders (DC/TMD) [21]. In some studies [21,22], the PSS-10 questionnaire was also used to assess stress levels. In general, the conclusion is that the use of an app such as BruxApp significantly increases each participant’s awareness of their bruxism habits, thereby raising the self-reported prevalence [13,15,18] and triggering a biofeedback effect that reduced bruxism throughout the week [14,19,20,23]. When using EMA, bruxism increased on weekdays, linking it to academic stress [20,21,22].

Portable EMG provided and objective assessment of masticatory muscle activity during wakefulness [12]. Only one study demonstrated high reliability of EMG for diagnosing waking bruxism, using short 15 min tests.

## 4. Discussion

As regards diagnostic criteria, diagnosing awake bruxism remains challenging, as the phenomenon of waking bruxism can range from a harmless behaviour to a risk factor for the development of muscle or myofascial pain, temporomandibular disorders, tooth wear and/or restorative problems [24,25]. This systematic review confirms that the diagnosis of AB remains a field undergoing methodological transition and lacks a gold standard diagnostic method, as while there are multiple tools available, none of them, *per se*, simultaneously meets the criteria for biological validity, clinical feasibility and epidemiological reproducibility.

Furthermore, diagnosis cannot rely solely on a single method. As each approach captures a different aspect of the phenomenon: self-perception, Ecological Momentary Assessment (EMA), associated clinical signs, or objective muscle activity. In this regard, the studies analysed converge on a central idea: simple self-report captures the patient’s subjective perception, making it useful for initial screening; EMA records behaviours in real time and in the natural environment, while also improving ecological validity; clinical examination provides context regarding signs and symptoms, increasing clinical suspicion and highlighting possible associated consequences; electromyography (EMG/sEMG) is the most objective method, recording current muscle activity, although diagnostic standardisation remains an unresolved issue. Furthermore, integrative models such as STAB, BruxScreen, ABIT (Bio-Intraoral Therapeutic Device) and, in particular, the EMA + EMG combination, represent the most promising approaches for a more robust assessment of AB [24,25,26], especially among such a vulnerable group as adolescents and young adults.

Methods based on self-assessment demonstrate clear clinical utility due to their simplicity, speed, low cost and ease of implementation, as is the case with the BruxScreen, although they have a major structural weakness in that they rely on the patient’s awareness of a behaviour that is often intermittent, fluctuating, partially perceived or even unnoticed. This is clearly evident in studies using portable EMG, where a significant proportion of subjects with no awareness or perception of waking bruxism were classified as bruxists, while other subjects who did perceive themselves as such showed no activity consistent with bruxism in that objective recording [26].

The most recent evidence, which compares the OBC (Oral Behaviour Checklist) and the EMA, also supports this limitation. Saracutu et al. suggest that the questionnaire may serve as a general screening tool, but not as a substitute for a more detailed behavioural assessment [19].

Ecological Momentary Assessment (EMA), now carried out using smartphones, represents the main advance in non-instrumental methods, as it reduces recall bias by requiring behaviour to be recorded in real time and within the individual’s usual environment [19,20,27]. In the studies reviewed, EMA made it possible to distinguish between different behaviours within the bruxism spectrum, such as tooth contact, clenching and grinding. For example, in patients with masticatory muscle pain, clenching was much more common than in healthy controls, and the likelihood of bruxism was almost five times higher [27].

Several studies suggest that the monitoring process itself can increase self-awareness and thus promote behavioural self-regulation, making EMA not only a diagnostic tool but also a potentially therapeutic one. The reduction in AB behaviours was greater when digital tools—which are so widely accepted by teenagers and young adults—were combined with traditional reminders [19].

Although EMA represents a major methodological advance, it remains a subjective self-report tool that relies on participant compliance, adequate understanding of the terminology and sustained engagement: it improves the “when” of reporting but does not fully resolve the problem of “what” is being perceived. Studies on BruxApp and similar tools themselves warn that prior instruction is important for improving understanding of the terms, although the reactivity effect must be taken into account; that is, the act of responding repeatedly may alter the observed behaviour itself. Therefore, the EMA substantially improves ecological validity but does not fully resolve the issue of objective validation of the AB [19,27,28].

EMA, particularly when smartphone-based, represents an important advance over retrospective self-report because it captures behaviours in real time and in the individual’s natural environment, thereby reducing recall bias [20,27]. However, EMA remains dependent on participant awareness, adherence, and understanding of the terminology used. Moreover, repeated monitoring may itself increase awareness and modify the behaviour being recorded, introducing a potential reactivity effect. Thus, although EMA improves ecological validity, it does not provide an entirely objective assessment of awake bruxism and should be interpreted in conjunction with other diagnostic information [20,27,28].

In healthy young adults, the frequency of episodes varies considerably depending on operational definitions, prior training and adherence to the protocol [19]. This variability, which does not invalidate the EMA, indicates that AB is sensitive to the measurement framework. Multicentre studies show that providing patients with prior instruction improves their understanding of terms such as “dental contact”, “occlusal pressure” or “mandibular thrust”, thereby improving data quality [19].

Ambulatory sEMG is currently considered the closest approach to a definitive instrumental assessment according to the international consensus. Its main advantage is that it allows for the magnitude and temporal distribution of activity to be quantified, as well as enabling the identification of subgroups with higher muscle load. In recent studies, the combination of EMA + sEMG has shown greater discriminatory power than either method alone in identifying probable AB and its relationship with symptoms of temporomandibular disorders [29]. In research practice, this multi-method convergence enhances internal validity: EMA provides behavioural ecology and sEMG provides physiological objectivity. However, sEMG also has limitations: artefacts caused by movement or speech, positive thresholds that are not entirely consistent across protocols, cost, the learning curve, and analytical infrastructure. Furthermore, the relationship between the number of events and clinical severity is not always linear [30]. This means we must avoid simplistic interpretations such as “higher EMG readings = worse patient” and instead consider psychosocial, pain-related and functional variables. In this regard, integrative tools such as STAB (Standardised Tool for the Assessment of Bruxism) are particularly valuable because they bring together subjective, clinical and instrumental domains within a single operational framework [29].

The clinical examination remains an important component, although its direct diagnostic value is more limited than has traditionally been assumed. Its greatest utility appears to lie in detecting associated signs and potential consequences of bruxism, rather than in demonstrating current activity. The STAB model (Standardised Tool for the Assessment of Bruxism) and related tools such as BruxScreen and ABIT (Bio-Intraoral Therapeutic Device) continue to regard the clinical examination as an essential component, but one that is subordinate to an integrated interpretation [30].

The clinical examination remains an important of the assessment, although its direct diagnostic value appears limited. Its main role is to identify signs and potential consequences associated with bruxism rather than to demonstrate current activity. For example, tooth wear showed very low sensitivity in identifying active bruxism compared with sEMG, although it had relatively high specificity; this suggests that its absence may support the absence of activity, but its presence does not prove current active bruxism. Similarly, ABIT records dental wear only as supplementary data and not as an identifying criterion [30,31].

However, buccal mucosal retraction showed a statistically significant association with a higher frequency of AB behaviours in adolescents assessed using EMA, suggesting that certain soft tissue signs may better reflect recent functional load than dental wear [19,20]. Even so, these clinical signs are neither specific nor sufficient on their own, as they may be influenced by other oral behaviours, the coexistence of sleep bruxism, or anatomical and occlusal factors. Consequently, clinical examination appears useful for raising diagnostic suspicion and establishing a “probable” diagnosis, but not for confirming AB on its own [19,20].

Portable or wearable EMG provides a more direct and objective measurement of masticatory muscle activity during wakefulness than self-reported or clinical assessment methods. The included studies demonstrated several potential advantages, including acceptable technical validity compared with stationary systems, the ability to record muscle activity under unrestricted conditions, and the potential to detect activity that may not be perceived by the individual [24,25,26,32]. However, these advantages should be interpreted with caution, as standardised diagnostic thresholds for awake bruxism have not yet been established.

Furthermore, partial recordings of 2.5–3 h can accurately reflect the activity pattern over a full day [26], and certain short tasks, particularly reading, demonstrate high test-retest reliability and very high specificity for confirming positive findings [17], which offers a clear practical advantage for research and clinical practice. Yamaguchi et al. noted that portable and wearable devices made it possible to record activity under unrestricted conditions and with performance levels close to those of stationary systems, although they expressly pointed out that there was still no definitive cut-off point for AB [24].

However, the greater objectivity provided by EMG in measuring muscle activity has not yet translated into universally accepted diagnostic criteria for AB. The review of portable devices had already noted that there was no definitive cut-off point for waking bruxism [24], and more recent studies retain this limitation, whether by using convenience thresholds, proposing preliminary cut-off points, or acknowledging that inter- and intra-individual variability remains considerable [12,17,25]. Subsequent studies reinforce this dual interpretation: on the one hand, EMG demonstrates the ability to detect activity not perceived by the subject, while there are still no universally standardised criteria.

The situation becomes even more complex when the measurement conditions are analysed. More recently, Angkulmahasuk et al. [12] proposed a strategy involving short 15 min tasks, demonstrating that the reading task exhibited high test–retest reliability, a moderate-to-high correlation with prolonged recordings, and 100% specificity, albeit with limited sensitivity. In other words, the short EMG test could be useful as a confirmatory test, but not as a stand-alone screening tool [12,26].

Nakagawa et al. showed that there were no significant differences between days, but there was considerable day-to-day variation. The indices for the number of episodes per hour and bursts per hour were the most stable, while the integral variables showed greater dispersion. This finding is crucial, as it suggests that any future diagnostic criteria will need to take intra-individual variability into account and will likely need to adjust their thresholds according to the severity and number of days recorded [17].

The most promising approach in the reviewed literature is the integration of methods. The combination of EMA + EMG specifically aims to bridge the gap between subjective perception and muscle recording. The study by Asami et al. proposed a preliminary cut-off point of 3.2 events/h using EMG at 20% MVC and with a duration of ≥1 s, with an AUC of 0.77, sensitivity of 0.69 and specificity of 0.76, which constitutes a promising approach for screening [25]. Conceptually, this combined model appears more robust than the isolated use of either tool because EMA helps to contextualise the behavioural significance of the EMG event, while EMG lends objectivity to the subjective report. However, the study itself acknowledges significant methodological limitations: the assessment of EMG events was restricted to time windows around EMA responses, true episodes may have been missed, and there is still no definitive standard for determining how many positive EMA records are sufficient [25]. Therefore, although this approach is probably the most promising of those reviewed, it should still be regarded as preliminary rather than definitive.

The new technologies and the research tools mentioned above —such as the experimental use of mobile apps (BruxApp) for EMA and the development of instruments such as ABIT, STAB and BruxScreen—represent important methodological advance in capturing daytime bruxism more objectively [22]. These integrative approaches accurately reflect the current conceptual shift, as waking bruxism should not be reduced to a binary question or a single clinical sign, but rather understood as a behavioural continuum that requires the integration of different sources of information [25,30].

The BruxScreen was designed as a brief screening tool, specifically to be practical, affordable and accessible in general practice, with short completion times and good comprehensibility; however, its developers themselves acknowledge that it still requires more in-depth psychometric validation and that it does not, on its own, resolve the issue of cut-off points [27]. The validation study comparing non-instrumental tools with sEMG showed that, for waking bruxism, specificity was acceptable, but sensitivity was modest, meaning that the non-instrumental approach may fail to identify a considerable proportion of cases [30].

ABIT, for its part, proposes a paediatric “AB spectrum” that introduces a “spectrum” approach of particular interest for adolescents, although it is currently in the pilot phase [20].

The methodological approach to initial screening should be comprehensive: a questionnaire/EMA for suspected cases and behavioural phenotyping, a clinical assessment for contextualisation, and EMG to objectively assess muscle activity [12,19,20,25]. In short, each method has its pros and cons, as shown in Table 3.

There are some limitations. Research on adolescent bruxism is limited by the lack of standardised diagnostic criteria and assessment methods, leading to inconsistent prevalence estimates. Most studies are cross-sectional and rely on subjective self- or parent reports, making it difficult to establish causality or ensure accurate diagnosis. Objective measurements are rarely used, important confounding factors are not always controlled, and clinical outcomes are assessed inconsistently. Therefore, more longitudinal studies with standardised and objective methods are needed.

## 5. Conclusions

There is currently no universally accepted gold standard for the diagnosis of awake bruxism in adolescents and young adults. The available evidence indicates that no single assessment method is sufficient to capture the complexity of this condition. Instead, a multimodal approach combining self-report questionnaires, Ecological Momentary Assessment, clinical examination, and portable electromyography appears to provide the most comprehensive diagnostic strategy.

Beyond dentistry, awake bruxism should be regarded as a multidisciplinary condition with potential implications for psychology, psychiatry, neurology, sleep medicine, physiotherapy, and pain management. A better understanding of awake bruxism and the implementation of reliable diagnostic strategies may contribute to more comprehensive patient care and foster collaboration among healthcare professionals involved in the management of orofacial pain and related disorders.

Future research should focus on validating objective assessment methods, establishing internationally accepted diagnostic criteria, and conducting longitudinal studies to clarify the natural history and clinical significance of awake bruxism in younger populations. From a clinical perspective, these findings may help dental practitioners and other healthcare professionals select and combine assessment methods according to the clinical context, using self-report and EMA for initial screening and behavioural characterisation, clinical examination to identify associated signs and consequences, and portable EMG as an objective complementary tool when further assessment is required.

## Figures and Tables

**Table 2:** Characteristics and diagnostic approaches of studies using non-instrumental methods for awake bruxism assessment.

Author, Year (Country)	Study Population and Age	Definition of Bruxism Used	Recorded Parameters	Assessment Method	Technical Assessment Details	Bruxism Outcomes
Colonna 2020 (Italy) [13]	-Young adult population -Mean age 24.2 ± 4.1 years. N = 103	-Study based on real-time self-report (possible bruxism).	Five real-time oral conditions were assessed: relaxed jaw muscles, tooth contact, tooth clenching, tooth grinding, and jaw bracing.	The study mainly used smartphone-assisted EMA). The DC/TMD criteria were also applied only as a screening tool to exclude participants with temporomandibular pain.	EMA/App: The BruxApp^®^ application was used. The software sent 20 random daily alerts with the aim of completing 7 days of valid recording. The recording time was from 8:00 to 12:30 and from 14:30 to 22:00. Participants had to respond within a maximum of 5 min after the alert. A success threshold of at least 60% of alerts answered per day was defined (minimum 12 of 20).	-Habit awareness: The authors emphasise that this strategy has high potential for collecting data on the spectrum of awake bruxism behaviours in the patient’s natural environment, overcoming the recall bias of traditional questionnaires.
Colonna et al., 2025 (Italy) [14]	-Young adult population. -Mean age 24.0 ± 0.8 years. N = 77	-Possible bruxism: Definition based on real-time self-report (EMA).	Five specific oral conditions were recorded: relaxed jaw muscles, jaw bracing, tooth contact, tooth clenching, and tooth grinding.	Smartphone-assisted EMA was used. The DC/TMD criteria were also applied exclusively as a screening protocol to exclude participants with temporomandibular pain.	EMA/App: The BruxApp application was used. Twenty random daily alerts were sent during three sessions of 7 days each, with three-month intervals between sessions (total follow-up of 6 months). Alerts were issued between 8:00–12:30 and 14:30–22:00. Subjects had to respond within a maximum of 5 min; otherwise, the response was marked as an error. A rate of 60% valid responses per day (minimum 12 answered alerts) was required to validate the day.	-Habit awareness: A slight increase in the report of “relaxed muscles” was observed (from 72.9% in the first session to 80.8% in the third), suggesting that prolonged monitoring may generate a biofeedback effect (EMI) and increase self-awareness of jaw habits.
Dias et al., 2021 (Portugal) [15]	-Young adult population -Mean age 24 ± 3.5 years N = 33	-Possible awake bruxism: Based on both real-time and retrospective patient self-report.	Five states of the jaw musculature during wakefulness were assessed: relaxed, bracing (tension without tooth contact), tooth contact, tooth clenching, and grinding.	A combination of EMA via a smartphone application and the OBC questionnaire was used.	-EMA/App: The BruxApp application was used during three consecutive 7-day periods with one-month intervals between them. The app sent 20 random daily alerts (between 7:30 and 22:00), and subjects had to respond within a maximum of 5 min; at least 12 valid responses per day were required. -Questionnaire: The OBC was used, a validated scale included in the Diagnostic Criteria for Temporomandibular Disorders (DC/TMD), which quantifies the frequency of oral behaviours over the previous month.	-Habit awareness: Use of the application significantly increased participants’ awareness of their habits, increasing the self-reported prevalence of bracing and clenching in the questionnaires after monitoring. -Correlation: A moderate positive correlation (+0.509) was found for bracing and a weak correlation (+0.380) for tooth contact between the final questionnaire and the last monitoring period.
Flores et al., 2024 (Brazil) [16]	-Young adult population. -Ages between 20 and 35 years. N = 150	-Possible bruxism: Based on self-report through EMA.	Five real-time conditions were recorded: relaxed jaw muscles, tooth contact, tooth clenching, tooth grinding, and jaw bracing. Muscle sensitivity parameters were also measured, such as pressure pain threshold (PPT) and conditioned pain modulation (CPM).	Smartphone-assisted EMA was used. In addition, the DC/TMD Pain Screener was used as an exclusion criterion, and quantitative sensory testing (QST) was performed using a digital algometer.	-EMA: The Mentimeter platform was used to send links through WhatsApp. Ten random daily alerts were sent for 7 consecutive days (5 weekdays and one weekend). Participants had to respond immediately. The links had a random expiration time of 5, 10, or 15 min. A minimum response rate of 60% was considered adequate. The actual mean response rate was 76.4%. -QST: PPT was measured bilaterally in the anterior temporalis and masseter muscles with an algometer with a 1 cm^2^ circular tip.	-Habit awareness: A significant increase in pressure pain threshold (PPT) values was observed after the study week. The authors attribute this to “EMA reactivity”, whereby constant monitoring increases self-awareness and allows participants to identify and avoid potentially harmful behaviours. -Correlation: No significant relationship was found between the frequency of bruxism behaviours and muscle sensitivity or the effectiveness of endogenous analgesia in these healthy adults.
Prado et al., 2023 (Brazil) [18]	Adolescent population Mean age 14.3 ± 1.5 years N = 403	-Possible: Based on the adolescent’s positive self-report. -Probable: Based on self-report plus clinical inspection of signs and symptoms.	-Awake bruxism: Tooth grinding and tooth clenching. -Sleep bruxism: Grinding, jaw bracing, and mandibular thrusting. -Clinical signs: Pain on palpation (masseter and temporalis muscles), presence of linea alba, tongue indentations, and severity of tooth wear.	-Questionnaire: Self-report for adolescents and parents. -Clinical examination: Physical inspection of intraoral and extraoral signs.	-Questionnaire: Dichotomous and frequency questions about the previous 14 days, with response options: “no”, “sometimes”, and “frequently”. A question about frequent headaches (more than 3 times per week) was included. -Clinical examination: Performed by a trained and calibrated examiner (inter-examiner kappa: 0.85; intra-examiner kappa: 0.80). -Tooth wear: A 5-point ordinal scale (0 to 4) was used, ranging from “no wear” to “loss of ≥2/3 of crown height”. -Pain: Assessed by bilateral pressure with the index and middle fingers on the masseter and temporalis muscles.	-Variability of the “probable” diagnosis: Depending on which clinical signs were combined with self-report, prevalence varied dramatically from 0.2% to 99.8% for awake bruxism, demonstrating high inconsistency in current criteria. -Awareness and associations: Frequent headaches and muscle pain on palpation showed a significant association with self-report of both types of bruxism. Self-report was considered essential, as many adolescents show clinical signs without being aware of the habit and vice versa.
Saracutu et al., 2025 (Italy) [19]	-Young adult population. -Mean age 22.5 ± 2.5 years. N = 100	-Possible bruxism: The study was based on retrospective and real-time self-report.	Four specific behaviours of masticatory muscle activity were assessed: tooth contact, jaw bracing, tooth clenching, and tooth grinding.	Two self-report methods were compared: smartphone-assisted Ecological Momentary Assessment (EMA) and a retrospective questionnaire (OBC).	-EMA: The research version of the BruxApp application was used. The protocol lasted 7 days with 20 random daily alerts distributed across time blocks to avoid meals (8:00–12:00, 15:00–19:00, 21:00–22:00). Participants had to respond within a maximum of 5 min, and 60% valid daily responses (12 of 20) were required to validate the report. -Questionnaire: Four items from the Oral Behaviour Checklist (OBC) were used, a validated tool that uses a 5-point Likert scale (from “never” to “all the time”) based on recall over the previous month.	-Correlation and awareness: A weak-to-moderate correlation (r = 0.3 to 0.55) was found between the two methods. Jaw bracing was the behaviour with the highest correlation level (0.55), whereas grinding had the lowest (0.23). Habit awareness: The authors suggest that EMA provides more detailed reports and is less affected by recall bias than the OBC questionnaire. In addition, real-time monitoring may help patients become more aware of their habits as part of cognitive behavioural therapy.
Schappo et al., 2023 (Brazil) [20]	-Adolescent population. -Mean age 16.9 ± 0.54 years N = 66	Probable awake bruxism (the study combined self-report with the presence of clinical signs such as indentations in the oral mucosa).	-Five oral behaviours: Tooth contact, tooth clenching, grinding, jaw bracing, and relaxed muscles. -Clinical signs: Presence or absence of indentations on the tongue, cheeks (buccal mucosa), and lips.	EMA method was used for behaviours, and a visual clinical examination was used for indentations.	-EMA: Conducted through WhatsApp (standard version). Messages were sent 15 times per day for 7 consecutive days (Monday to Sunday). Messages were sent randomly between 8:00 and 19:00. Students had to respond immediately or up to a maximum of 10 min later. -Clinical examination: Performed by a single trained investigator under biosafety standards and natural light to identify indentations.	-Association and awareness: A significant association was found between a higher frequency of awake bruxism and the presence of cheek indentations. In addition, clenching, grinding, and jaw bracing were more frequent on weekdays than during the weekend, suggesting a link with academic stress and a possible improvement in habit awareness (biofeedback) through use of the EMA method.
Stanisic et al., 2024 (Sweden) [21]	-10 young adults (23 to 30 years)	-Possible bruxism: Definition based on real-time self-report (EMA).	Five specific oral conditions were recorded: relaxed jaw muscles, bracing without tooth contact, light tooth contact, tooth clenching, and grinding.	A combination of EMA through a mobile application and several self-administered questionnaires was used.	-EMA / App: The Swedish version of the BruxApp application was used. Twenty-five daily alerts were sent randomly during two seven-day periods (a baseline and a repeat one month later). Users had to record their state within 5 min after the alert. A minimum of 12 valid responses per day was required to include the day in the analysis. -Questionnaires: Brux-5Q (bruxism screening), 3Q/TMD (temporomandibular disorder screening), OBC-19 (Oral Behaviour Checklist for daytime parafunctions), and PSS-10 (Perceived Stress Scale) were used.	-Habit awareness: Participants reported a significant increase in jaw bracing in the questionnaires during the second observation period, which was attributed to greater self-awareness generated by use of the app and prior educational information. -Correlation with stress: A moderate positive correlation was found between perceived stress (PSS-10) and awake bruxism activity (r = 0.54, *p* = 0.017).
Zani et al., 2021 (Italy) [23]	-Young adult population -Mean age: 22.9 ± 3.2 years N = 30	-Possible bruxism: Study based on real-time self-report.	Five specific oral conditions were measured: -Relaxed jaw muscles. -Tooth contact. -Tense jaw. -Tooth clenching. -Tooth grinding.	A smartphone application-assisted EMA strategy was used.	EMA/App: The BruxApp application was used. The software sent 20 daily alerts at random times. Recording lasted for 7 consecutive days. Subjects had to respond within 5 min after the alert; otherwise, the response was marked as an error. A minimum of 60% valid responses per day was required; if this threshold was not reached, the software automatically added one extra recording day until seven valid days were completed.	-Habit awareness: A slight decrease in the frequency of bruxism behaviours was observed over the week, suggesting that EMA monitoring increases self-awareness and may function as an intervention (biofeedback).

Note: EMA (Ecological Momentary Assessment); OBC (Oral Behaviour Checklist) questionnaire; DC/TMD (diagnostic criteria for temporomandibular disorders); Brux-5Q (bruxism screening), 3Q/TMD (temporomandibular disorder screening), OBC-19 (Oral Behaviour Checklist for daytime parafunctions), and PSS-10 (Perceived Stress Scale).

**Table 3:** Characteristics of studies assessing awake bruxism using electromyographic methods.

Author, Year (Country)	Study Population and Age	Definition of Bruxism Used	Recorded Parameters	Assessment Method	Technical Assessment Details	Bruxism Outcomes
Angkulmahasuk et al., 2025 (Japan) [12]	-Adult population. -Mean age 28.0 ± 3.1 years N = 34.	-Definite (or instrumental) bruxism: Uses electromyography (EMG) to objectively measure muscle activity.	Electromyographic activity of the masseter muscle was measured, assessing: -Average number of EMG bursts per hour. -Burst duration. -Peak burst value relative to maximum voluntary contraction (%MVC). -Integral activity value (µV.s and %MVC.s). -Subjective perception through the OBC questionnaire.	Surface EMG was used with a portable recorder and the self-administered OBC questionnaire.	-EMG: A lightweight device (EMG Logger FLA-500-SD, GC Co. Ltd.) was placed on the masseter muscle of the habitual chewing side. -Recording protocol: Short-duration recordings (15 min) were conducted during four specific tasks (silent reading, bead manipulation with chopsticks, watching relaxing videos, and simple mathematics tasks), and a long -duration recording (7 h) was conducted during normal daily activities (reference standard). -Questionnaire: A partial version of the OBC (POBC) was used, scoring 6 specific items associated with awake bruxism behaviours.	-Awareness and self-report: A lack of significant correlation was found between OBC questionnaire scores and objective EMG parameters. Participants who reported a low frequency of habits in the questionnaire showed high levels of actual muscle activity. -Validity of short tests: The 15-min reading task proved to be the most reliable, with 100% specificity and a positive predictive value of 1, suggesting that it can be used clinically to confirm the diagnosis of awake bruxism in a less burdensome way than all-day recordings.
Nakagawa et al., 2025 (Japan) [17]	-Young adult population. -Mean age 24.1 ± 0.9 years N = 91.	-Definite bruxism: Uses EMG.	Masseter muscle activity during the day was measured using five indices: number of episodes per hour, number of bursts per hour, number of bursts with intensity ≥20% of maximum voluntary contraction (MVC) per hour, burst integral value per hour, and standardised burst integral value per hour.	Surface EMG was used with a portable wearable device and a self-report questionnaire to determine habit awareness.	-EMG: The FLA-500-SD device (Furusawa Lab Appliance Co.), an ultraminiature data logger, was placed at the centre of the masseter muscle on the habitual chewing side. Measurements were performed for at least 8 h per day on three different days within a two-week period. Recording included normal daily activities and at least two meals. Processing: MVC calibrations were performed at the beginning and end of each session. Meal periods and signal noise were excluded from the analysis. -Questionnaires: A written binary-response questionnaire (“yes/no”) was used to assess awareness of tooth clenching during the day.	-Habit awareness: Forty-eight subjects were aware of clenching their teeth and 43 were not. However, there were no significant differences in EMG parameters between the two groups, and substantial overlap in the frequency of EMG waves was observed between them. -Relationship with intensity: A significant negative correlation was found between the number of episodes and the coefficient of variation; in other words, subjects with a higher burst frequency tended to show more stable and less variable activity between days.

Note: EMG: electromyography; MVC: maximum voluntary contraction; OBC: Oral Behaviour Checklist; POBC: Partial Oral Behaviour Checklist.

## Data Availability

No new data were created or analyzed in this study. Data sharing is not applicable to this article.

## Supplementary Materials

The following supporting information can be downloaded at: https://www.mdpi.com/article/10.3390/dj14090606/s1, Table S1: PRISMA 2020 Checklist.
