# Supplementary material for: Diagnosis of Awake Bruxism in Adolescents and Young Adults: A Systematic Review
Source: Dent J (Basel). 2026 Sep 18;14(9):606. doi: 10.3390/dj14090606 (PMC13605587; doi:10.3390/dj14090606)
Supplement: Supplementary file 1 [file dentistry-14-00606-s001.zip › dentistry-4457227-supplementary.pdf]

# PRISMA 2020 Checklist

## Diagnosis of Awake Bruxism in Adolescents and Young Adults: A Systematic Review

*Completed against the submitted manuscript. “Not reported” indicates that the information could not be identified in the manuscript; “not applicable” is used where the item does not apply to the qualitative synthesis.*

| Section and topic | Topic | Item # | Checklist item | Location where item is reported |
|---|---|---|---|---|
| TITLE | Title | 1 | Identify the report as a systematic review. | Title. |
| ABSTRACT | Abstract | 2 | See the PRISMA 2020 for Abstracts checklist. | Abstract. Note: the abstract reports the objective, information sources/date, risk-of-bias tools, main results and conclusion; registration/funding are not stated in the abstract. |
| INTRODUCTION | Rationale | 3 | Describe the rationale for the review in the context of existing knowledge. | Introduction, paragraphs describing prevalence and the range/limitations of available diagnostic approaches. |
| INTRODUCTION | Objectives | 4 | Provide an explicit statement of the objective(s) or question(s) the review addresses. | End of Introduction; Methods, Section 2.3 (PECOS framework). |
| METHODS | Eligibility criteria | 5 | Specify the inclusion and exclusion criteria for the review and how studies were grouped for the syntheses. | Methods, Section 2.3, Article selection and eligibility criteria. |
| METHODS | Information sources | 6 | Specify all databases, registers, websites, organisations, reference lists and other sources searched or consulted, and the date when each source was last searched or consulted. | Methods, Section 2.1: PubMed/MEDLINE, SciELO, Scopus, Cochrane and Web of Science; searched 17 March 2026. |
| METHODS | Search strategy | 7 | Present the full search strategies for all databases, registers and websites, including any filters and limits used. | Methods, Section 2.2 gives one search string stated as used across all databases. Database-specific syntax, filters/limits are not reported. |
| METHODS | Selection process | 8 | Specify the methods used to decide whether a study met the inclusion criteria, including how many reviewers screened each record/report, whether they worked independently, and any automation tools used. | Partially reported in Methods, Section 2.1: MPPL and NPG performed study selection, with JARR resolving discrepancies. Independence and automation tools are not explicitly stated. |
| METHODS | Data collection process | 9 | Specify the methods used to collect data from reports, including number/independence of reviewers, procedures for obtaining/confirming data, and any automation tools used. | Partially reported in Methods, Section 2.1: data extraction was carried out by MPPL and NPG, with JARR for discrepancies. Detailed extraction procedure, independence and automation are not reported. |
| METHODS | Data items | 10a | List and define all outcomes for which data were sought. Specify whether all compatible results were sought and, if not, the methods used to decide which results to collect. | Partially reported in Section 2.3 (PECOS: correlation of the method and awareness of the habit) and Tables 2–3 (recorded parameters/bruxism outcomes). No explicit statement that all compatible results were sought. |
| METHODS | Data items | 10b | List and define all other variables for which data were sought and describe assumptions made about missing or unclear information. | Study characteristics are presented in Tables 2–3 (country, population/age, definition, parameters, method, technical details, outcomes), but assumptions about missing/unclear information are not reported. |
| METHODS | Study risk of bias assessment | 11 | Specify the methods used to assess risk of bias in included studies, | Methods, Section 2.4: JBI for cross-sectional |

| Section and topic | Topic | Item # | Checklist item | Location where item is reported |
|---|---|---|---|---|
| | | | including tool(s), number/independence of reviewers, and any automation tools used. | studies, RoB 2 for randomized trials and QUADAS-2 for diagnostic accuracy studies; two reviewers (MPPL, NPG), disagreements resolved with JARR. |
| METHODS | Effect measures | 12 | Specify for each outcome the effect measure(s) used in the synthesis or presentation of results. | Not explicitly specified as a review-level method. The review is primarily descriptive/narrative; individual correlations, specificity, predictive values and other measures are reported where available. |
| METHODS | Synthesis methods | 13a | Describe the processes used to decide which studies were eligible for each synthesis. | Studies at high risk of bias were excluded from the qualitative synthesis (Section 2.4); remaining studies were grouped by main diagnostic approach in Tables 2 and 3. |
| METHODS | Synthesis methods | 13b | Describe any methods required to prepare the data for presentation or synthesis, such as handling missing summary statistics or data conversions. | Not reported / not applicable to the descriptive qualitative synthesis as presented. |
| METHODS | Synthesis methods | 13c | Describe any methods used to tabulate or visually display results of individual studies and syntheses. | Results, Section 3.3: characteristics are presented in separate tables according to non-instrumental versus EMG-based assessment. No additional plotting method is described. |
| METHODS | Synthesis methods | 13d | Describe any methods used to synthesize results and provide a rationale for the choice(s). If meta-analysis was performed, describe the model, heterogeneity methods and software. | A qualitative/narrative synthesis is presented, but the synthesis method and rationale are not explicitly described in the Methods. No meta-analysis was performed. |
| METHODS | Synthesis methods | 13e | Describe any methods used to explore possible causes of heterogeneity among study results. | No formal subgroup/meta-regression analysis. Methodological heterogeneity is described narratively in Results and Discussion. |
| METHODS | Synthesis methods | 13f | Describe any sensitivity analyses conducted to assess robustness of synthesized results. | Not applicable. |
| METHODS | Reporting bias assessment | 14 | Describe any methods used to assess risk of bias due to missing results in a synthesis (reporting biases). | Not applicable. |
| METHODS | Certainty assessment | 15 | Describe any methods used to assess certainty (or confidence) in the body of evidence for an outcome. | Methods, Section 2.4: formal GRADE assessment was not performed because of substantial methodological heterogeneity; rationale is provided. |
| RESULTS | Study selection | 16a | Describe the results of the search and selection process, from records identified to studies included, ideally using a flow diagram. | Results, Section 3.1 and Figure 1: 418 records identified, 196 duplicates removed, 222 screened, 30 full-text reports assessed, 13 studies included; 11 retained in qualitative synthesis after risk-of-bias assessment. |
| RESULTS | Study selection | 16b | Cite studies that might appear to meet inclusion criteria but were excluded, and explain why they were excluded. | Reasons for 17 full-text exclusions are summarized in Section 3.1, but the individual excluded reports are not cited/listed. |
| RESULTS | Study characteristics | 17 | Cite each included study and present its characteristics. | Results, Section 3.3; Tables 2 and 3. |
| RESULTS | Risk of bias in studies | 18 | Present assessments of risk of bias for each included study. | Results, Section 3.2 and Table 1. |
| RESULTS | Results of individual studies | 19 | For all outcomes, present for each study summary statistics and an effect estimate with precision where appropriate. | Tables 2 and 3 present study-specific bruxism outcomes and available quantitative results. Precision measures are not consistently |

| Section and topic | Topic | Item # | Checklist item | Location where item is reported |
|---|---|---|---|---|
| | | | | available/reported because of heterogeneous study designs and outcomes. |
| RESULTS | Results of syntheses | 20a | For each synthesis, briefly summarise the characteristics and risk of bias among contributing studies. | Results, Sections 3.2–3.3 and Tables 1–3; narrative summary by diagnostic approach. |
| RESULTS | Results of syntheses | 20b | Present results of all statistical syntheses conducted. If meta-analysis was done, present summary estimate and precision and measures of heterogeneity. | No meta-analysis/statistical synthesis was performed. Narrative results are presented in Section 3.3 and Discussion. |
| RESULTS | Results of syntheses | 20c | Present results of all investigations of possible causes of heterogeneity among study results. | No formal investigation performed; methodological heterogeneity is described narratively (Section 3.3 and Discussion). |
| RESULTS | Results of syntheses | 20d | Present results of all sensitivity analyses conducted to assess robustness of synthesized results. | Not applicable. |
| RESULTS | Reporting biases | 21 | Present assessments of risk of bias due to missing results (reporting biases) for each synthesis assessed. | Not applicable. |
| RESULTS | Certainty of evidence | 22 | Present assessments of certainty (or confidence) in the body of evidence for each outcome assessed. | Not assessed. Section 2.4 explains why formal GRADE assessment was not performed. |
| DISCUSSION | Discussion | 23a | Provide a general interpretation of the results in the context of other evidence. | Discussion, Sections discussing self-report, EMA, clinical examination, portable EMG and multimodal approaches. |
| DISCUSSION | Discussion | 23b | Discuss any limitations of the evidence included in the review. | Discussion, limitations paragraph: lack of standardized diagnostic criteria, cross-sectional designs, subjective reports, limited objective measures, confounding and inconsistent clinical outcomes. |
| DISCUSSION | Discussion | 23c | Discuss any limitations of the review processes used. | Partially reported in the limitations paragraph, mainly limitations of the evidence base. Review-process limitations (e.g., search restrictions, selection/extraction limitations) are not clearly separated or discussed. |
| DISCUSSION | Discussion | 23d | Discuss implications of the results for practice, policy, and future research. | Discussion and Conclusions: multimodal diagnostic approach; need for objective validation, internationally accepted criteria and longitudinal studies; clinical guidance on combining self-report/EMA, examination and EMG. |
| OTHER INFORMATION | Registration and protocol | 24a | Provide registration information for the review, including register name and registration number, or state that the review was not registered. | Methods, opening paragraph: PROSPERO registration CRD420251181371. |
| OTHER INFORMATION | Registration and protocol | 24b | Indicate where the review protocol can be accessed, or state that a protocol was not prepared. | Methods, opening paragraph: PROSPERO registration CRD420251181371. |
| OTHER INFORMATION | Registration and protocol | 24c | Describe and explain any amendments to information provided at registration or in the protocol. | Not reported. |
| OTHER INFORMATION | Support | 25 | Describe sources of financial or non-financial support for the review, and the role of funders or sponsors. | Funding statement: 'This research received no external funding.' |
| OTHER INFORMATION | Competing interests | 26 | Declare any competing interests of review authors. | Conflicts of Interest statement: authors declare no conflicts of interest. |
| OTHER INFORMATION | Availability of data, code and | 27 | Report which review materials are publicly available and where they | Data Availability Statement: 'On request in |

| Section and topic | Topic | Item # | Checklist item | Location where item is reported |
|---|---|---|---|---|
| | other materials | | can be found (e.g., forms, extracted data, analysis data, code, other materials). | corresponding author.' Supplementary Materials list Table S1 (Data extraction) and Table S2 (Different methods). Public availability of forms/code is not specified. |

*Source: PRISMA 2020 statement (Page MJ et al., BMJ 2021;372:n71). Checklist completed for the manuscript supplied by the authors.*
